# Sensitivity of *VHL* mutant kidney cancers to HIF2 inhibitors does not require an intact p53 pathway

**DOI:** 10.1073/pnas.2120403119

**Published:** 2022-03-31

**Authors:** Laura A. Stransky, Sean M. Vigeant, Bofu Huang, Destiny West, Thomas Denize, Emily Walton, Sabina Signoretti, William G. Kaelin

**Affiliations:** ^a^Department of Medical Oncology, Dana–Farber Cancer Institute, Harvard Medical School, Boston, MA 02215;; ^b^Department of Pathology, Brigham and Women’s Hospital, Harvard Medical School, Boston, MA 02115;; ^c^HHMI, Chevy Chase, MD 20815

**Keywords:** ccRCC, HIF, *VHL*, TP53, belzutifan

## Abstract

Inactivation of the *VHL* tumor suppressor gene is the signature initiating event in clear cell renal cell carcinoma (ccRCC), which is the most common form of kidney cancer. The VHL tumor suppressor protein marks hypoxia-inducible factor 1 (HIF1) and HIF2 for proteasomal degradation when oxygen is present. The inappropriate accumulation of HIF2 drives tumor formation by VHL tumor suppressor protein (pVHL)–defective ccRCC. Belzutifan, a first-in-class allosteric HIF2 inhibitor, has advanced to phase 3 testing for advanced ccRCC and is approved for ccRCCs arising in patients with VHL disease, which is caused by germline *VHL* mutations. HIF2 can suppress p53 function in some settings and preliminary data suggested that an intact p53 pathway, as measured by activation in response to DNA damage, was necessary for HIF2 dependence. Here, we correlated HIF2 dependence and p53 status across a broader collection of ccRCC cell lines. We also genetically manipulated p53 function in ccRCC lines that were or were not previously HIF2-dependent and then assessed their subsequent sensitivity to HIF2 ablation using CRISPR-Cas9 or the HIF2 inhibitor PT2399, which is closely related to belzutifan. From these studies, we conclude that p53 status does not dictate HIF2 dependence, at least in preclinical models, and thus is unlikely to be a useful biomarker for predicting which ccRCC patients will respond to HIF2 inhibitors.

## Significance

*VHL* tumor suppressor gene inactivation is a hallmark of clear cell renal cell carcinoma (ccRCC), the most common form of kidney cancer, and promotes tumor growth by stabilizing the hypoxia-inducible factor 2 (HIF2) transcription factor. HIF2 inhibitors appear to be helpful for some, but not all, ccRCC patients in clinical trials. Previous preclinical and clinical data suggested that only ccRCCs that can activate the p53 tumor suppressor in response to DNA damage would respond to HIF2 inhibitors. Here, we show that an intact p53 pathway is neither necessary nor sufficient for the sensitivity of ccRCCs to HIF2 inhibitors, suggesting that it would be premature to use p53 status to determine which ccRCC patients should be treated with a HIF2 inhibitor.

Inactivation of the VHL tumor suppressor protein (pVHL) is the usual initiating, or truncal, event in clear cell renal cell carcinoma (ccRCC) (1–7), which is the most common form of kidney cancer. pVHL is the substrate recognition component of an E3 ubiquitin ligase that targets the alpha subunits of the heterodimeric HIF (hypoxia-inducible factor) transcription factor for destruction when oxygen is plentiful. In the absence of pVHL, deregulation of HIF, in particular HIF2, drives the growth of ccRCC (8–13).

Drugs that inhibit the HIF2-responsive growth factor VEGF (vascular endothelial growth factor) are now mainstays of ccRCC treatment (14). However, HIF2 controls hundreds of other genes, including additional genes that are known or suspected of playing pathogenic roles in ccRCC (15, 16). Although HIF2 was classically viewed as undruggable, Richard Bruick and Kevin Gardner identified compounds that can bind to a previously unappreciated pocket in HIF2α and, in so doing, induce an allosteric change in HIF2α that prevents it from binding to its partner protein ARNT and hence to DNA (17–20). These compounds were optimized further by Peloton Therapeutics to produce the HIF2 inhibitors PT2399 (preclinical compound), the first-generation clinical compound PT2385, and the second-generation compound PT2977 (now called MK-6482 or belzutifan) (21–25).

Both PT2385 and PT2977 display significant activity when used to treat ccRCC patients who have failed standard-of-care agents such as VEGF inhibitors and immune checkpoint inhibitors (25–28). However, many heavily pretreated ccRCC patients do not respond to these drugs and most that do respond eventually progress (25–28). Consistent with these observations, some *VHL* mutant ccRCC cell lines are not dependent on HIF2 (21). There is a pressing need to understand the molecular basis of the HIF2 independence exhibited by some ccRCC cell lines and some ccRCC patients.

We previously identified an acquired p53 mutation in a HIF2-independent subclone of the HIF2-dependent ccRCC cell line 786-O and Brugarolas and coworkers identified a p53 mutation, R273H, in a HIF2-independent ccRCC tumor in a ccRCC patient who had a mixed response to PT2385, suggesting that p53 mutations caused resistance (21, 28). Moreover, in our initial survey of ccRCC cell lines, we noted that an intact p53 pathway, as measured by induction of p53 and p21 after DNA damage, was necessary (although not sufficient) for HIF2 dependence (21). Notably, HIF2 suppression can enhance p53 activation, at least in response to radiation, suggesting that p53 activation could contribute to the antiproliferative effects of HIF2 inhibitors (29). In this study, we examined an expanded panel of ccRCC cell lines and genetically manipulated *TP53* in ccRCC cells to address whether p53 status is, indeed, an important determinant of HIF2 dependence and hence sensitivity to pharmacological HIF2 inhibitors. Our data suggest that p53 activity is neither necessary nor sufficient for dependence on HIF2, at least in the context of ccRCC.

## Results and Discussion

**Fig. 1.**
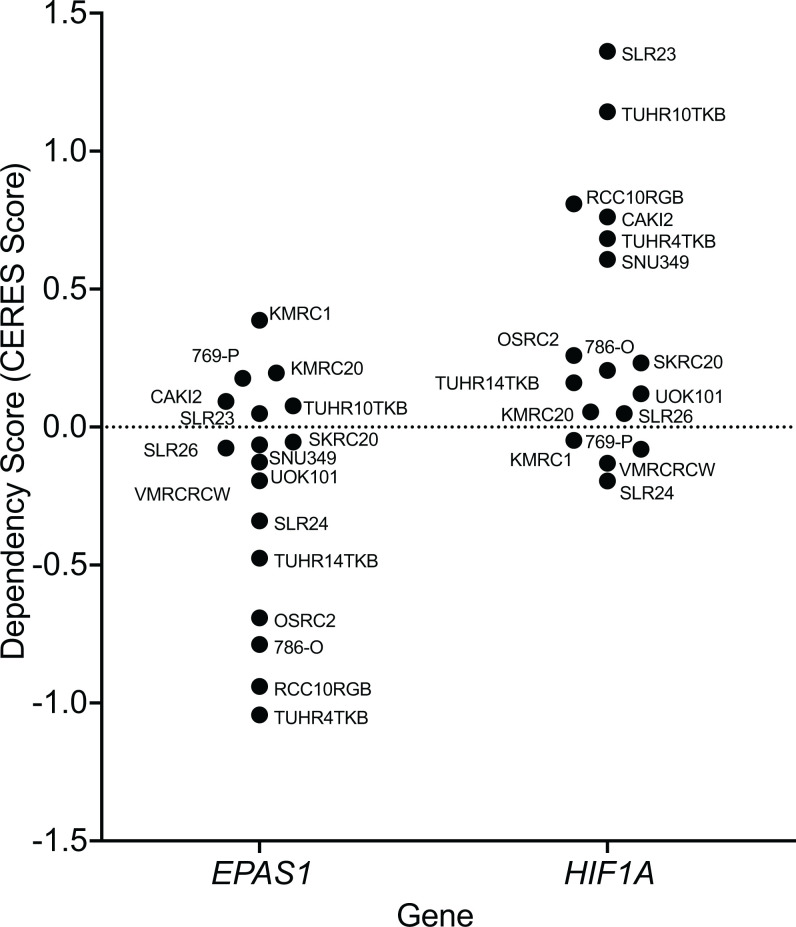
HIF1α and HIF2α dependence varies across *VHL* mutant ccRCC cell lines based on genome-wide CRISPR-Cas9 screens. *EPAS1* and *HIF1A* dependency (CERES) scores of *VHL* mutant ccRCC cell lines from the DepMap database of genome-wide CRISPR-Cas9 dependency screens. CERES scores estimate gene dependency, taking into account the copy number–related fitness effects of CRISPR-Cas9 genome editing.

We first examined the Broad Institute DepMap dataset (https://www.depmap.org) with respect to the depletion of *EPAS1* single-guide RNAs (sgRNAs) over time in ccRCC cell lines that had been subjected to genome-wide CRISPR-Cas9 screens. *EPAS1* sgRNAs were significantly depleted in some ccRCC lines, including TUHR14TKB, OSRC2, 786-O, RCC10RGB, and TUHR4TKB, indicative of HIF2 dependence, but not in many other ccRCC lines, such as CAKI2, 769-P, and SLR23, suggestive of HIF2 independence (Fig. 1). The sensitivity of the 786-O, CAKI2, and 769-P cells to the *EPAS1* sgRNAs is consistent with our earlier studies (21). Notably, *HIF1A* sgRNAs were variably enriched in ccRCC lines included in the DepMap dataset, consistent with the earlier suggestion that HIF1α acts to constrain ccRCC growth (11, 12, 30, 31) (Fig. 1).

**Fig. 2.**
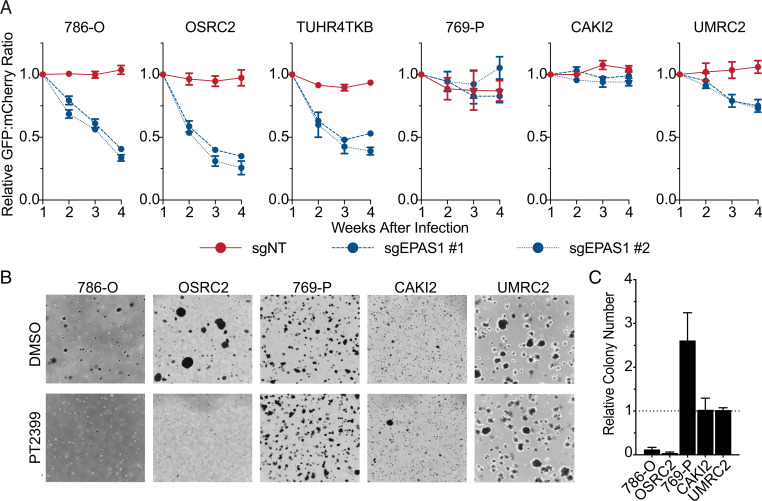
HIF2α dependence varies across *VHL* mutant ccRCC cell lines based on cellular competition and anchorage-independent growth assays. (*A*) Relative ratios of mixtures of cells expressing 1) GFP and either a nontargeting control sgRNA (sgNT) or the indicated sgRNA targeting *EPAS1*, or 2) mCherry and sgNT, over time, as determined by flow cytometry. Ratios were normalized such that the ratio at *T* = 1 wk after lentiviral introduction of the reporters and sgRNAs was 1. Data represent the mean ± SEM of at least two independent replicates. (*B*) Representative photomicrographs of soft agar colonies formed by the indicated *VHL* mutant ccRCC cell lines that were grown in the presence of 2 µM PT2399 or DMSO vehicle control. (*C*) Quantification of soft agar colonies formed by the indicated *VHL* mutant ccRCC cell lines as in *B*. Data represent mean ± SEM of at least two independent replicates.

To corroborate this variable HIF2 dependence, we performed low-throughput competition assays with selected ccRCC lines that we infected to produce either a control sgRNA and mCherry or one of two different *EPAS1* sgRNAs (or the same control sgRNA) and green fluorescent protein (GFP) (*SI Appendix*, Fig. S1*A*), aiming for an ∼1:1 ratio of mCherry-positive cells and GFP-positive cells. We then passaged the cells for 4 weeks and monitored the GFP:mCherry ratio by fluorescence-activated cell sorting (FACS). The *EPAS1* sgRNA cells were dramatically depleted in the HIF2-dependent lines 786-O, OSRC2, and TUHR4TKB, but much less so in the HIF2-independent lines 769-P and CAKI2, and in UMRC2 cells, which we reported before are also largely HIF2-independent (21) (Fig. 2*A*). Similar results were obtained with both *EPAS1* sgRNAs and when the mCherry and GFP fluorescent proteins were swapped in the sgRNA expression vectors and the mCherry:GFP ratio was monitored (*SI Appendix*, Fig. S2*B*). Moreover, PT2399 suppressed soft agar growth by the HIF2-dependent lines 786-O and OSRC2 but not by the HIF2-independent lines 769-P, CAKI2, and UMRC2 (Fig. 2 *B* and *C*). TUHR4TKB did not form soft agar colonies and therefore could not be studied in this assay. PT2399 reproducibly enhanced the soft agar growth of 769-P cells, although the significance of this finding is not clear. These findings, together with our earlier study, confirm that the HIF2 dependence of ccRCC lines is variable, which provides an opportunity for studying de novo and acquired resistance to HIF2 inhibitors.

**Fig. 3.**
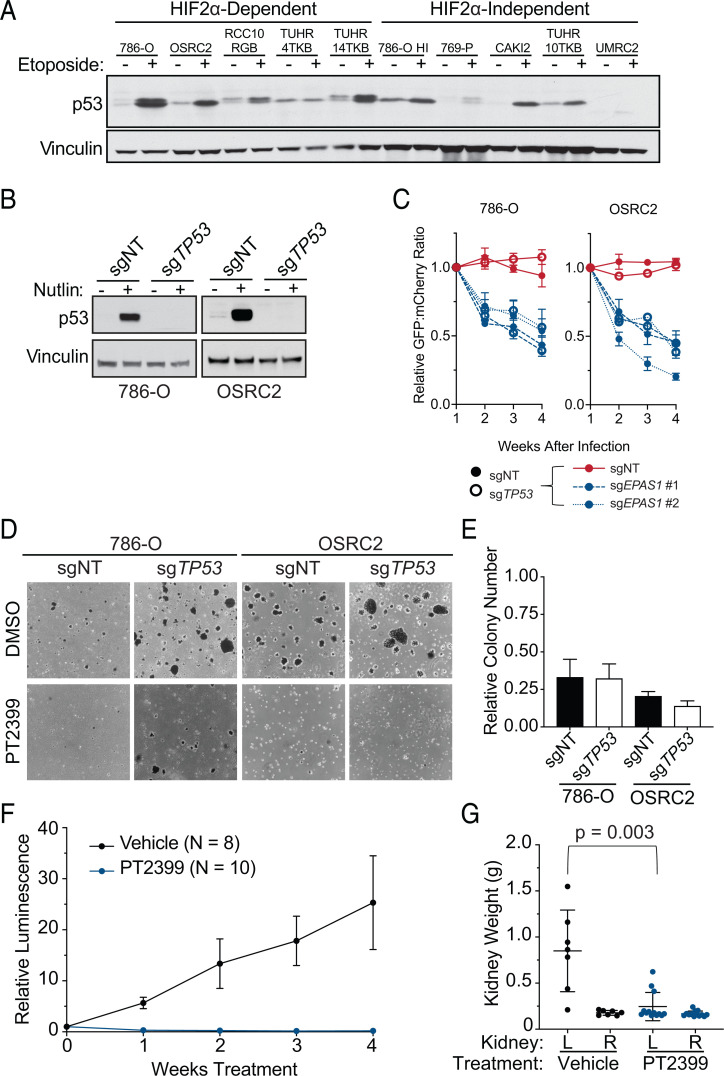
WT *TP53* is not required for HIF2α dependence of *VHL* mutant ccRCC lines. (*A*) Immunoblots of the indicated *VHL* mutant ccRCC cell lines that were (+) or were not (-) treated with 10 µM etoposide for 24 h. (*B*) Immunoblots of isogenic pairs of 786-O and OSRC2 cells that underwent CRISPR-Cas9–based gene editing with the indicated sgRNAs and then treatment with 10 µM Nutlin for 24 h where indicated. (*C*) Relative ratio of mixtures of cells from *B* (filled circles, cells expressing nontargeting sgRNA; open circles, cells expressing sg*TP53*) that were then engineered to express 1) GFP and either a nontargeting control sgRNA (sgNT) or the indicated sgRNA targeting *EPAS1*, or 2) mCherry and sgNT, over time, as determined by flow cytometry. Ratios were normalized such that the ratio at *T* = 1 wk was 1 after lentiviral introduction of the reporters and sgRNAs. Data represent the mean ± SEM of at least two independent replicates. (*D*) Representative photomicrographs of soft agar colonies formed by 786-O and OSRC2 cells as in *B* that were grown in the presence of 2 µM PT2399 or DMSO vehicle control. (*E*) Quantification of soft agar colonies formed by the cell lines as in *D*. Shown are mean colony numbers in the presence of 2 µM PT2399 relative to DMSO. Data are mean ± SEM of at least two independent replicates. (*F*) Average relative BLI intensity over time of orthotopic xenografts (left kidney) formed by 786-O that stably expressed firefly luciferase and in which *TP53* was inactivated using CRISPR-Cas9 (sg*TP53*). The tumor-bearing mice were treated daily with 30 mg/kg PT2399 or vehicle control by oral gavage. For each mouse, BLI readings were normalized to the pretreatment BLI value for that mouse. (*G*) Kidney weights at necropsy for the mice studied in *F*. L, left; R, right.

We next measured the induction of p53 by the DNA-damaging agent etoposide in a panel of ccRCC cell lines, expanding upon the number of lines we interrogated previously (21) (Fig. 3*A*). As we reported before, p53 was robustly induced in the HIF2-dependent 786-O cells but not in the HIF2-independent 769-P and UMRC2 cells (21). On the other hand, p53 was not induced in the HIF2α-dependent line TUHR4TKB but was induced in the HIF2α-independent line CAKI2. Therefore, an intact p53 pathway appears to be neither necessary nor sufficient for HIF2α dependence.

To investigate this further, we repeated the competition and soft agar assays using 786-O and OSRC2 cells after first inactivating p53 using CRISPR-Cas9. We confirmed that Nutlin treatment induced p53 in 786-O cells expressing a control sgRNA but not a *TP53* sgRNA (Fig. 3*B*). Loss of p53 did not prevent the pharmacodynamic effects of PT2399, as measured by down-regulation of Cyclin D1 (*SI Appendix*, Fig. S2*A*). Moreover, eliminating p53 did not significantly mitigate the competitive disadvantage of cells expressing *EPAS1* sgRNAs nor did it rescue soft agar growth in the presence of PT2399, despite increasing soft agar growth in the absence of PT2399 (Fig. 3 *C*–*E* and *SI Appendix*, Fig. S2*B*). Finally, 786-O cells lacking *TP53* remained sensitive to PT2399 in orthotopic tumor assays in vivo, as determined by reduced tumor size and Cyclin D1 and Ki67 expression, in mice treated with PT2399 compared with vehicle (Fig. 3 *F* and *G* and *SI Appendix*, Fig. S2 *C–E*).

**Fig. 4.**
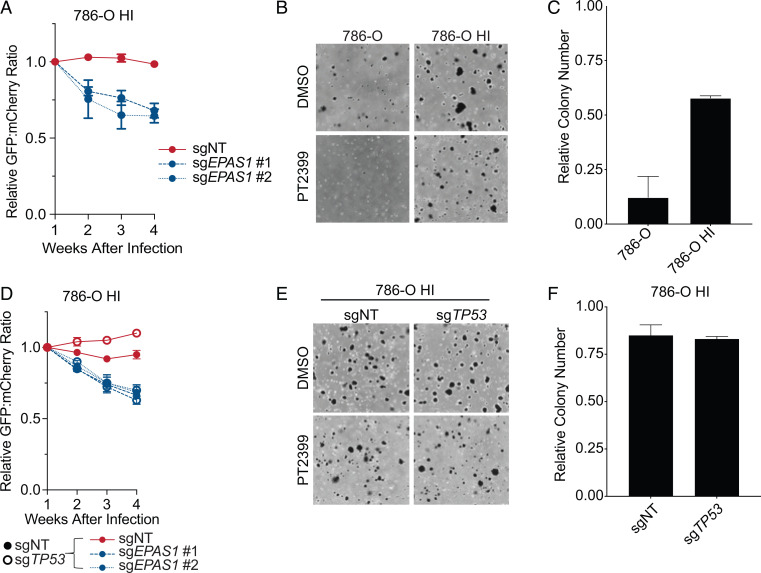
*TP53* mutation is not required for the HIF2α independence of 786-O HI cells. (*A*) Relative ratios of mixtures of 786-O HI cells expressing 1) GFP and either a nontargeting control sgRNA (sgNT) or the indicated sgRNA targeting *EPAS1*, or 2) mCherry and sgNT, over time, as determined by flow cytometry. Ratios were normalized such that the ratio at *T* = 1 wk after lentiviral introduction of the reporters and sgRNAs was 1. Data represent the mean ± SEM of at least two independent replicates. (*B*) Representative photomicrographs of soft agar colonies formed by 786-O and 786-O HI cells that were grown in the presence of 2 µM PT2399 or DMSO vehicle control. (*C*) Quantification of soft agar colonies formed by the cell lines in *B*. Shown are mean colony numbers in the presence of 2 µM PT2399 relative to DMSO. Data are mean ± SEM of at least two independent replicates. (*D*) Relative ratio of mixtures of 786-O HI cells (filled circles, cells expressing nontargeting sgRNA; open circles, cells expressing sg*TP53*) that were then engineered to express 1) GFP and either a nontargeting control sgRNA (sgNT) or the indicated sgRNA targeting *EPAS1*, or 2) mCherry and sgNT, over time, as determined by flow cytometry. Ratios were normalized such that the ratio at *T* = 1 wk after lentiviral introduction of the reporters and sgRNAs was 1. Data represent the mean ± SEM of at least two independent replicates. (*E*) Representative photomicrographs of soft agar colonies formed by 786-O HI cells as in *D* that were grown in the presence of 2 µM PT2399 or DMSO vehicle control. (*F*) Quantification of soft agar colonies formed by the cell lines as in *D*. Shown are mean colony numbers in the presence of 2 µM PT2399 relative to DMSO. Data are mean ± SEM of at least two independent replicates.

We previously identified a 786-O subclone that spontaneously acquired partial HIF2 independence (“786-O HI”) relative to parental 786-O cells (21), which we reconfirmed in competition and soft agar assays (Fig. 4 *A–C* and *SI Appendix*, Fig. S3 *D* and *E*). These cells, in contrast to parental 786-O, have high basal p53 levels and do not further induce p53 in response to DNA damage (Fig. 3*A*). Hence, they are phenotypically p53 mutant. Based on sequencing a limited number of complementary DNA (cDNA) clones from these cells, we previously genotyped these cells as p53 R248W (21). To more rigorously genotype these cells, we performed a battery of assays. First, we confirmed that the 786-O HI cells were indeed derived from 786-O cells, as determined by short tandem repeat (STR) analysis (*SI Appendix*, Table S1). Next, we karyotyped 25 parental 786-O cells and 25 786-O HI cells. This analysis showed that 786-O are highly heterogeneous and, as reported before, nearly triploid, although some chromosomes are present in two, four, or five copies (*SI Appendix*, Table S2). Notably, these cells have four copies of chromosome 17, which harbors *TP53*. The 786-O HI cells appeared to be highly genomically unstable based on the presence of new isochromosomes (*SI Appendix*, Table S3).

We next determined *TP53* mutant allele frequencies by next-generation sequencing of genomic DNA. In the parental 786-O cells, we detected both wild-type (WT) and R248W *TP53* alleles at an ∼60:40 ratio. In the 786-O HI cells, we detected 49% P278A, 3% R248W, and 48% WT *TP53* (*SI Appendix*, Fig. S3*A*). Finally, we PCR-amplified *TP53* cDNA from these cells with primers designed to include codons 248 and 278. The PCR products were recombined into a plasmid vector, which was used to transform *Escherichia coli*. We then sequenced multiple individual cDNA clones. In the parental 786-O cells, 63% of the cDNAs were WT at codon 248 and 37% were R248W mutant at codon 248 out of 71 cDNAs sequenced. In 58 of these 71 cDNAs the sequence corresponding to codon 278 was also evaluable. No P278A cDNAs were detected. In the 786-O HI cells, 99% of the cDNAs were WT at codon 248 and 1% were R248W at codon 248 out of 71 cDNAs sequenced. In 70 of these 71 cDNAs the sequence corresponding to codon 278 was also evaluable; 8% of these cDNA were WT at codon 278 and 92% were P278A at codon 278. The R248W and P278A mutations were never found in *cis* and no other mutations were detected. In parallel, we only detected WT *TP53* sequences in 25 cDNAs from OSRC2 cells, of which 22 were evaluable for codon 278 in addition to codon 248 (*SI Appendix*, Fig. S3 *B* and *C*). These results indicate that our initial hypothesis that the resistance of 786-O HI to PT2399 was caused by the R248W mutation was incorrect and instead might be caused by the P278A mutation.

*TP53* mutations can be loss-of-function, dominant-negative, or neomorphic. The continued sensitivity of the 786-O and OSRC2 cells to PT2399 after CRISPR-Cas9–mediated elimination of *TP53* strongly suggested that pure loss-of-function or dominant-negative *TP53* mutations would not cause resistance to PT2399. To address the possibility that the P278A allele caused the PT2399 resistance of the 786-O HI cells through a neomorphic activity, we inactivated *TP53*, including the P278A allele, in the 786-O HI cells. These cells remained insensitive to PT2399 in competition and soft agar assays, suggesting that expression of the P278A variant is not necessary for the PT2399 insensitivity of the 786-O HI cells (Fig. 4 *D–F* and *SI Appendix*, Fig. S3*F*).

**Fig. 5.**
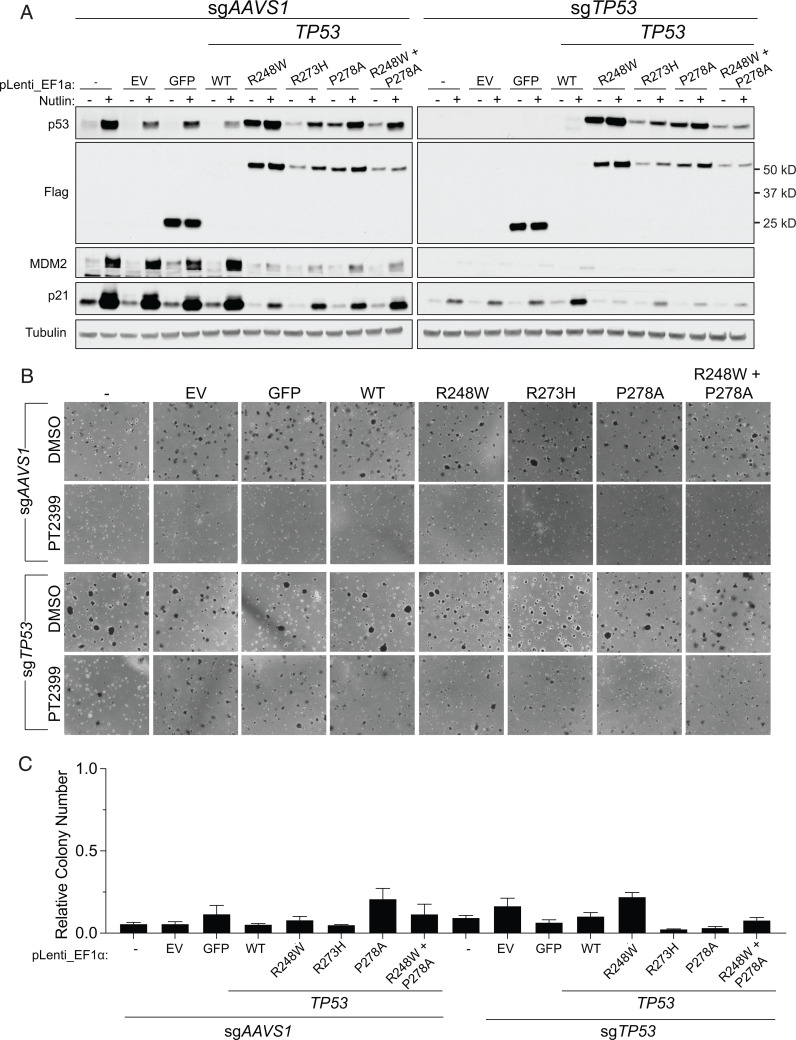
Expression of *TP53* mutations previously associated with HIF2α independence is not sufficient to confer HIF2α independence. (*A*) Immunoblots of 786-O cells expressing control sgRNA (sg*AAVS1*) or sg*TP53* that were then engineered to express GFP, the indicated p53 variants, or an empty vector (EV). The cells were (+) or were not (-) treated with 10 µM Nutlin for 24 h prior to harvest. (*B*) Representative photomicrographs of soft agar colonies formed by 786-O cells as in *A* that were grown in the presence of 2 µM PT2399 or DMSO vehicle control. (*C*) Quantification of soft agar colonies formed by 786-O cells as in *B*. Shown are mean colony numbers in the presence of 2 µM PT2399 relative to DMSO. Data are mean ± SEM of at least two independent replicates.

**Fig. 6.**
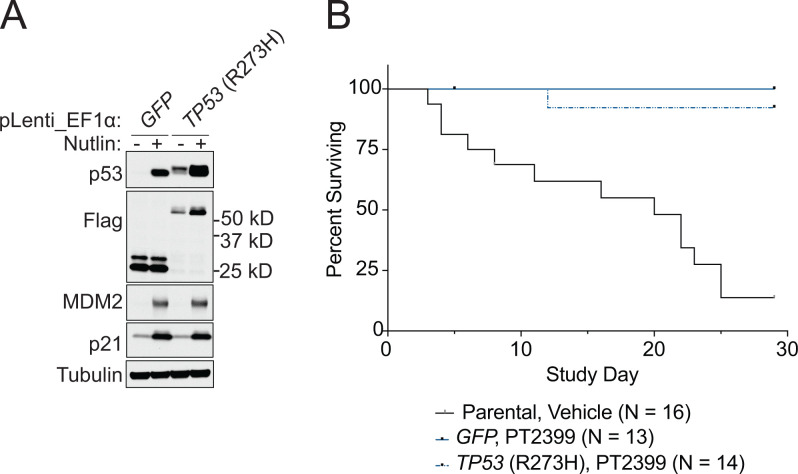
Expression of the TP53 R273H mutation previously associated with HIF2α independence is not sufficient to confer HIF2α independence. (*A*) Immunoblot of OSRC2-Fluc cells engineered to express GFP or *TP53* R273H and then treated (+) or not (-) with 10 µM Nutlin for 24 h. (*B*) Survival of mice bearing orthotopic xenografts (left kidney) formed by OSRC2 cells as in *A* or parental OSRC2. Tumor-bearing mice were treated daily with 30 mg/kg PT2399 once daily by oral gavage.

To address the issue of sufficiency, and to further probe for a possible neomorphic activity, we introduced exogenous WT p53, p53 R248W, p53 R273H, p53 R278A, GFP, or the empty backbone vector into 786-O and OSRC2 cells in which endogenous p53 was or was not first eliminated using CRISPR (Fig. 5*A* and *SI Appendix*, Fig. S4*A*). R248W was included because it is a well-characterized neomorphic p53 mutant that was mistakenly suspected of causing the resistance of the 786-O HI cells (see above), while the R273H mutant was detected in a kidney cancer patient who developed resistance to PT2399 (28). None of the exogenous p53 mutants caused pharmacodynamic resistance to PT2399, regardless of endogenous *TP53* status, as reflected by decreased accumulation of the HIF2-responsive gene products Cyclin D1 and NDRG1 (*SI Appendix*, Fig. S5) or resistance to PT2399 in soft agar assays (Fig. 5 *B* and *C* and *SI Appendix*, Fig. S4 *B* and *C*). Moreover, expression of R273H did not mitigate the survival benefit conferred by PT2399 in OSRC2 orthotopic tumor assays or prevent the suppression of the HIF2 target gene *CCND1* by the drug (Fig. 6 and *SI Appendix*, Fig. S6).

These data, collectively, strongly suggest that an intact p53 pathway is not required for the HIF2 dependence of *VHL* mutant ccRCC cells, despite early conjecture to the contrary, and also suggest that p53 mutants such as R273H that have been associated with PT2399 resistance do not necessarily cause that resistance. Although we interrogated promoters of various strengths to drive the expression of exogenous mutant p53, it remains formally possible that higher levels of expression would have caused resistance. Moreover, it remains possible that *TP53* status could alter the sensitivity of *VHL* mutant kidney cancers under conditions that were not captured by our preclinical models, such as in conditions created in an immunocompetent host. Nonetheless, our findings argue that it would be premature to use p53 status to exclude kidney cancer patients from trials of HIF2 inhibitors.

The prevalence of *TP53* mutations in ccRCC could be as low as 2 to 6% based on analyses primarily of primary nephrectomy specimens, although the true prevalence might be higher in advanced disease or after multiple rounds of therapy (1, 4, 32–37). In one large study that included over 400 samples, ∼15% of ccRCC metastases harbored *TP53* mutations (37). Another study found that 12% of ccRCC patient-derived xenografts (PDXs) bore *TP53* mutations, suggesting that *TP53* mutations increase ccRCC fitness and successful PDX propagation, both of which might correlate with more aggressive clinical disease (38). Moreover, other genes in the p53 pathway, such as *ATM* and *MDM2*, are also mutated, albeit infrequently, in ccRCC (1, 4, 32–35), and *TP53* loss cooperates with *VHL* loss to promote murine ccRCC (39, 40). Additionally, single specimens might be insufficient to capture the full complement of mutations present in a ccRCC tumor. For example, multiregion whole-exome sequencing showed that *TP53* mutation was subclonal in tumors from 4 out of 10 ccRCC patients examined (35). Notably, increased p53 protein levels, which are often indicative of a compromised p53 pathway, have been observed in up to 50% of ccRCCs and are associated with poor outcomes (41, 42). Therefore it is probable that our findings are relevant for a significant fraction of ccRCC patients who would be eligible for treatment with a HIF2 inhibitor.

## Materials and Methods

### Cell Lines and Cell Culture.

786-O, 769-P, CAKI2, and HEK293FT cells were obtained from the American Type Culture Collection. 786-O HI cells arose from a subculture of 786-O cells infected with empty pLenti6-HA backbone (21). OSRC2, TUHR4TKB, TUHR10TKB, TUHR14TKB, and RCC10RGB cell lines were obtained from the RIKEN BioResource Research Center Cell Bank. UMRC2 cells were a gift of Bert Zbar and Marston Linehan, National Cancer Institute, Bethesda, MD. 786-O, 786-O HI, TUHR4TKB, RCC10RGB, and UMRC2 cells were grown in Dulbecco’s modified Eagle’s medium (DMEM) (Gibco; 11995073). CAKI2 cells were cultured in McCoy’s medium 5A (Corning; 10050CV). OSRC2, TUHR10TKB, and TUHR14TKB were cultured in RPMI-1640 (Gibco; 11875119). Basal media were supplemented with 10% fetal bovine serum (FBS) (Gemini Bioproducts; 100-106), 100 U/mL penicillin, and 100 µg/mL streptomycin (Gibco; 25200056). Cells were maintained in a humidified incubator at 37 °C and 5% CO_2_.

Cas9-expressing cells were generated by infection with pLenti-EF1α-Cas9-Flag-IRES-Neo, a gift of Samuel McBrayer, W.G.K. laboratory, described in ref. 43.

### Chemicals.

PT2399 was provided by Peloton Therapeutics. Etoposide was purchased from APP Pharmaceuticals. Nutlin-3a was purchased from Selleck Chemicals.

### Plasmids.

#### CRISPR-Cas9 plasmids.

sgRNAs were cloned into pLentiCRISPRv2 (Addgene; 52961), pLentiCRISPR_zeo, a gift of Samuel McBrayer, described in ref. 43, or pLentiGuide (Addgene; 117986) modified to also express GFP or mCherry, gifts of Vidyasagar Koduri and Benjamin Lampson, W.G.K. laboratory. Vector backbones were linearized by *Bsm*BI digestion (New England Biolabs; R0580) in NEB buffer 3.1 for 2 h at 55 °C, followed by incubation at 80 °C for 20 min to inactivate the enzyme. The linearized vector backbone was purified by gel extraction using the QIAquick Gel Extraction Kit per the manufacturer’s instructions (Qiagen; 28706). sgRNA sense and antisense oligos were synthesized by IDT. Oligonucleotide pairs were mixed at an equimolar ratio and phosphorylated by phosphonucleotide kinase (New England Biolabs; M0201) and annealed by heating to 37 °C for 30 min, 95 °C for 5 min, and cooled to 25 °C at a rate of 5 °C/min in a thermocycler. Phosphorylated and annealed oligos were diluted 1:200 in DNase/RNase–free water (Corning; 46000CV). Diluted oligos and linearized vector were ligated with T4 DNA ligase (New England Biolabs; M0202) overnight at room temperature. A portion of the ligation reaction was transformed into chemically competent HB101 *E. coli* (Promega; L2011). Transformed bacteria were selected by plating on Luria-Bertani-agarose plates containing 150 µg/mL ampicillin and incubated at 30 °C overnight. Individual colonies were expanded in LB broth containing 100 µg/mL ampicillin in a 30 °C shaking incubator overnight. Plasmid DNA was extracted with the QIAprep Spin Plasmid Miniprep Kit (Qiagen; 27106) and validated by Sanger sequencing.

#### *sgRNA sequences, with* Bsm*BI cloning sequences.*

sg*EPAS1* no. 1 sense 5′-caccgAATCTCCTCATGGTCGCA-3′
sg*EPAS1* no. 1 antisense 5′-aaacTGCGACCATGAGATTc-3′
sg*EPAS1* no. 2 sense 5′-caccgTCATGAGGATGAAGTGCA-3′
sg*EPAS1* no. 2 antisense 5′-aaacTGCACTTCATCCTCATGAc-3′
sg*TP53* sense 5′-caccgCCATTGTTCAATATCGTCCG-3′
sg*TP53* antisense 5′-aaacGCGACGATATTGAACAATGGc-3′
sgNT135 sense 5′-caccgCGCTTCCGCGGCCCGTTCAA-3′
sgNT135 antisense 5′-aaacTTGAACGGGCCGCGGAAGCGc-3′

#### p53 expression vectors.

pDONR223 containing open reading frames (ORFs) encoding WT or R248W *TP53* including silent mutations to abrogate binding of the sg*TP53* described above was a gift of Andrew Giacomelli and William Hahn, Dana–Farber Cancer Institute. The QuikChange II XL Site-Directed Mutagenesis Kit (Agilent; 200522) was used per the manufacturer’s instructions to introduce P278A or R273H mutations into the sgRNA-resistant WT *TP53* ORFs using the primers listed below. Mutagenesis reactions were digested with *Dpn*I to remove template plasmids. A portion of the mutagenesis reaction was transformed into XL-10 Gold chemically competent *E. coli* (Agilent; 200315). Plasmids containing the desired mutations were isolated from spectinomycin-resistant transformants using the QIAprep Spin Plasmid Miniprep Kit (Qiagen; 27106) and validated by Sanger sequencing.

pDONR223 entry clones encoding N-terminally Flag-tagged p53 (WT or mutants) or enhanced GFP (EGFP) were generated by amplifying the *TP53* ORFs from 10 ng of the plasmids described above or *EGFP* from pLentiGuide_GFP with the primers listed below using KOD Xtreme HotStart Polymerase according to the manufacturer’s specifications (MilliporeSigma; 719753). The primers introduced DNA sequences encoding a Flag epitope tag and a flexible linker (GGGGS) onto the 5′ end of the *TP53* or *EGFP* ORFs and 12-bp-long truncated attB sites at the 5′ and 3′ ends of the ORFs. Amplification of a fragment of the expected size was confirmed by agarose gel electrophoresis. Full-length attB1 and attB2 sites were added by a second PCR with the attB extension primers listed below, and amplification of the fragment of the expected size was again confirmed by agarose gel electrophoresis. After each PCR, unincorporated primers and dNTPs were removed using the Monarch PCR and DNA Cleanup Kit (New England Biolabs; T1030).

To clone Flag-tagged inserts into pDONR223, 150 ng pDONR223 (Invitrogen) was mixed with 150 ng of the purified, full-length PCR insert and 1 µL BP clonase II (Invitrogen; 11789020). The total reaction volume was brought to 5 µL with TE buffer (10 mM Tris⋅HCl and 1 mM ethylenediaminetetraacetate [EDTA]). The BP reaction was incubated overnight at room temperature. Two microliters of the recombination reaction was transformed into 25 µL HB101 chemically competent *E. coli* (Promega; L2011), and spectinomycin-resistant transformants were isolated by growth on LB-agarose plates containing 50 µg/mL spectinomycin overnight at 30 °C. Individual colonies were expanded by growth in LB broth containing 50 µg/mL spectinomycin overnight at 30 °C with constant shaking. Plasmids were isolated with the QIAprep Spin Plasmid Miniprep Kit (Qiagen; 27106) and incorporation of the desired ORF was validated by Sanger sequencing.

Once validated, the ORFs were shuttled into the pLenti-EF1α-Gate-PGK-hygromycin destination vector, a gift of Gang Lu, W.G.K. laboratory, by LR recombination reactions. One hundred and fifty nanograms of each entry and destination vector was mixed with 1 µL LR clonase II (Invitrogen; 11791100) and the reaction volume was brought to 5 µL with TE buffer. Recombination reactions were allowed to proceed overnight at room temperature. Two microliters of the reactions was transformed into 25 µL HB101 chemically competent *E. coli* (Promega; L2011). Kanamycin-resistant transformants were isolated by growth on LB-agar plates containing 50 µg/mL kanamycin overnight at 30 °C. Individual colonies were expanded overnight at 30 °C in a shaking incubator in LB broth containing 50 µg/mL kanamycin. Plasmids were isolated using the QIAprep Spin Plasmid Miniprep Kit (Qiagen; 27106) and validated by Sanger sequencing. A pDONR223 containing only a short multiple cloning site within the attB-flanked region was also a gift of Gang Lu.

#### Targeted mutagenesis primers.

*TP53* R273H forward 5′-GCTTTGAGGTGCATGTTTGTGCCTG-3′
*TP53* R273H reverse 5′-CAGGCACAAACATGCACCTCAAAGC-3′
*TP53* P278A forward 5′-GTGCGTGTTTGTGCCTGTGCTGGGAGAGACCGGCGC-3′
*TP53* P278A reverse 5′-GCGCCGGTCTCTCCCAGCACAGGCACAAACACGCAC-3′

#### Primers to amplify Flag-tagged TP53 and EGFP for Gateway cloning.

attB12-Flag-GGGGS-*TP53* forward 5′-AAAAAGCAGGCTAAATGGACTACAAGGACGACGATGACAAGGGT
  GGCGGAGGGAGCGAGGAGCCGCAGTCAGATCC-3′
attB12-*TP53* reverse 5′-AGAAAGCTGGGTTTCAGTCTGAGTCAGGCCTT-3′
attB12-Flag-GGGGS-*TP53* EGFP forward 5′-AAAAAGCAGGCTAAATGGACTACAAGGACGACGATGACAA
  GGGTGGCGGAGGGAGCGTGAGCAAGGGCGAGGAGCT-3′
attB12-*EGFP* reverse 5′-AGAAAGCTGGGTTTCACTTGTACAGCTCGTCCAT-3′
attB1 extension forward 5′-GGGGACAAGTTTGTACAAAAAAGCAGGCT-3′
attB2 extension reverse 5′-GGGGACCACTTTGTACAAGAAAGCTGGGT-3′

### Generation of Lentivirus.

HEK293FT cells were seeded at a density of 45,000 cells per square centimeter in DMEM supplemented with 10% FBS, with the plate size determined by the amount of virus desired. The following day, the cells were cotransfected with the lentiviral plasmid of interest, psPAX2 (Addgene; 12260) and pMD2.G (Addgene; 12259), at a ratio of 2:1:1 using Lipofectamine 2000 transfection reagent at a ratio of 1 µg DNA:3 µL Lipofectamine (Life Technologies; 11668019). Plasmid DNA and Lipofectamine were diluted separately in Opti-MEM medium and incubated for 5 min at room temperature prior to transfection. Plasmid- and Lipofectamine-containing solutions were then mixed together vigorously and incubated at room temperature for 20 min before dropwise addition to the plates containing HEK293FT cells. The following day, media were replaced with DMEM supplemented with 30% FBS. Viral supernatants were collected 24 and 48 h later and pooled, spun at 1,200 × *g*, and filtered with 0.45-µm SCFA (surfactant-free cellulose acetate) filters (Corning; 431220) before aliquoting and storage at −80 °C.

### Generation of Stable Cell Lines by Lentiviral Infection.

Three hundred thousand cells per well were seeded into 6-well plates in 2.75 mL of the culture medium normally used for the recipient cell line. Two hundred and fifty microliters of viral supernatant was added per well such that each well received a different virus. One additional well received no virus to serve as an uninfected control. Plates were centrifuged at 4,000 rpm in an A-4-81 rotor for the 5810 R centrifuge (Eppendorf) for 30 min at 37 °C. Rotor buckets were rotated horizontally 180° after 15 min to evenly distribute cells within each well. Cells were incubated in a humidified chamber overnight at 37 °C and 5% CO_2_. Media containing virus were replaced with normal growth media the following day. The next day, selection antibiotic (1 µg/mL puromycin, 200 µg/mL hygromycin [except 25 µg/mL for OSRC2], 200 µg/mL G418, or 400 µg/mL zeocin as appropriate) was added to the growth media to select successfully infected cells. Cells were passaged in selection media in parallel with the uninfected control until no viable cells remained in the uninfected control, after which selection antibiotic was removed and cultures were maintained in normal growth media. Gene knockout or exogenous expression was confirmed by Western blotting as described below before proceeding to phenotypic assays.

### Western Blotting.

Adherent cells were rinsed once with ice-cold phosphate-buffered saline (PBS) (pH 7.4) and scraped in 500 µL of ice-cold PBS and transfered to 1.5 mL Eppendorf tubes. The cells were then pelleted by centrifugation at 1,200 × *g* for 5 min at 4 °C. After aspirating the supernatant, the cell pellets were lysed in EBC buffer (50 mM Tris, pH 7.5, 150 mM NaCl, 0.5% Nonidet P-40, and 1 mM EDTA) supplemented with cOmplete protease inhibitors (Roche; 11697498001) and PhosSTOP phosphatase inhibitors (Roche; 4906845001). Pellets were thoroughly disrupted by pipetting and rotated for 30 min at 4 °C. Lysates were cleared by centrifugation at 17,000 × *g* for 15 min at 4 °C. Protein concentration of the resulting whole-cell lysates was determined by the Bradford assay (Bio-Rad Laboratories; 5000006).

For Western blotting, 20 µg of total protein for each sample was diluted in Laemmli sample buffer, heated at 95 °C for 5 min, loaded into 4 to 15% Tgx polyacrylamide gels (Bio-Rad Laboratories; 4561086 or 5671085), separated by sodium dodecyl sulfate polyacrylamide gel electrophoresis, and transferred to nitrocellulose membranes with the Transblot Turbo System (Bio-Rad Laboratories; 1704271) according to the manufacturer’s specifications. The membrane was rinsed briefly with deionized water before transferred proteins were visualized with Ponceau S stain (Cell Signaling Technology; 59803) to ensure even transfer. Ponceau S stain was removed by two washes for 5 min in TBS-T (Tris-buffered saline [20mM Tris, 150 mM sodium chloride] with 0.1% Tween-20) with constant agitation. The membrane was then incubated for 1 h at room temperature in 5% nonfat milk in TBS-T to block nonspecific antibody binding with constant agitation. Milk was washed off the membrane by two 5-min washes in TBS-T. The membrane was incubated overnight with constant agitation at 4 °C with primary antibodies (listed below) diluted in TBS-T supplemented with 5% protease-free bovine serum albumin (Gold BioTechnology; A-420-500) and 0.05% sodium azide. The following day, the primary antibody solution was removed, and the membrane was washed three times with TBS-T for 10 min. Washed membranes were incubated with horseradish peroxidase (HRP)–conjugated secondary antibodies (goat anti-mouse immunoglobulin G [IgG] [Jackson ImmunoResearch Laboratories; 115-035-003] or goat anti-rabbit IgG [Jackson ImmunoResearch Laboratories; 111035003]) diluted 1:5,000 in TBT-T containing 5% nonfat milk for 1 h at room temperature with constant agitation. The secondary-antibody solution was removed and the membrane was washed three times with TBS-T for 10 min. Bound antibodies were detected with enhanced chemiluminescent HRP substrates SuperSignal West Pico PLUS Chemiluminescent Substrate (Thermo Scientific; 34578), Immobilon Western Chemiluminescent HRP Substrate (MilliporeSigma; WBKLS0500), or Pierce ECL Plus Western Blotting Substrate (ThermoFisher; 32106). Chemiluminescent signal was detected with autoradiographic film (Denville; E3031).

#### Primary antibodies.

Rabbit anti-HIF2α, Cell Signaling Technologies (7096), used at 1:1,000
Mouse anti-p53, Cell Signaling Technologies (2524), used at 1:1,000
Rabbit anti–Cyclin D1, Cell Signaling Technologies (2978), used at 1:1,000
Rabbit anti-NDRG1, Cell Signaling Technologies (5196), used at 1:1,000
Rabbit anti-p21, Cell Signaling Technologies (2947), used at 1:20,000
Rabbit anti-MDM2, Cell Signaling Technologies (86934), used at 1:1,000
Rabbit anti-Flag, Cell Signaling Technologies (14793), used at 1:1,000
Mouse anti-vinculin, Sigma (V9131), used at 1:100,000
Mouse anti-tubulin, Sigma (T5168), used at 1:10,000

### Cellular FACS-Based Competition Assays.

Viruses delivering nontargeting sgRNAs or sgRNAs targeting *EPAS1* in the pLentiGuide_GFP or mCherry backbone were titrated in Cas9-expressing 786-O cells to determine the volume of virus per well to infect ∼10% of cells as determined by antibiotic resistance. For competition assays, Cas9-positive cell lines were infected with a mixture of a GFP-expressing lentivirus and an mCherry-expressing lentivirus in amounts designed to infect 10% of the cells with each virus based on the titration data above. Successfully infected cells were selected with 1 µg/mL puromycin until no cells remained in an uninfected control treated in parallel (typically 2 to 3 d). Initial population abundance, measured as the percentage of live cells showing GFP or mCherry positivity, was assessed 1 wk after infection by flow cytometry using an LSRFortessa cell analyzer (BD Biosciences; 649225) with BD FACSDiva software. Population abundance over time was assessed by weekly flow cytometry on the same instrument. Relative abundance was calculated as the ratio of GFP:mCherry or mCherry:GFP as indicated in the figure legends and normalized to the same ratio at the initial time point.

### Colony Formation in Soft Agar.

Soft agar assays for all cell lines were carried out in non–tissue culture–treated 6-well plates (Corning; 351146), with the exception of 786-O, which was seeded in ultralow-adhesion 6-well plates (Corning; 3471). A 3% (weight/volume) SeaPlaque agarose (Lonza; 50100) solution was prepared in PBS (pH 7.4) and sterilized by autoclaving. This stock solution was stored at room temperature, melted before use by gentle microwaving, and maintained in liquid form in a 50 °C water bath during assay setup. Warmed 3% agarose stock solution was diluted 1:2 with growth media to obtain a 1% agarose solution. Two milliliters of 1% agarose was added to each well of the 6-well plate and allowed to solidify at room temperature for at least 20 min. After solidifying, 1 mL of a 0.4% agarose solution containing 50,000 cells and 2 µM PT2399 or dimethyl sulfoxide (DMSO) vehicle control was gently added to each well. The cell-containing layer was allowed to solidify at room temperature for 1 h, after which 1.5 mL of growth media containing 2 µM PT2399 or DMSO vehicle control was added to each well. Plates were incubated in a humidified incubator maintained at 37 °C and 5% CO_2_. Overlay media were changed twice per week until macroscopic colonies formed (2 to 5 wk depending on the cell line). At the termination of the assay, media were aspirated and replaced with 1 mL 0.1% iodonitrotetrazolium chloride (Sigma-Aldrich; I8377) in PBS for 48 h to stain colonies. For each treatment condition to be tested, cells were plated in triplicate and the entire assay was repeated for each cell line at least twice.

Plates were scanned with a desktop scanner (Epson; Perfection V700 photo scanner). Colonies were quantified with Fiji ImageJ (44). Microscopic images were obtained with the Revolve microscope (Discover Echo).

### Orthotopic Xenografts.

All experimental procedures related to orthotopic xenografts were approved by the Institutional Animal Care and Use Committee of Dana-Farber Cancer Institute. 786-O and OSRC2 cells were infected with the pLL3.7-EF1α-Fluc-Neo vector, a gift from Matthew Oser, W.G.K. laboratory, described in ref. 43, encoding firefly luciferase to facilitate tumor monitoring by bioluminescent imaging (BLI). Female Ncr nude mice (Taconic; NCRNU-F) were anesthetized by intraperitoneal injection of 140 mg/kg ketamine and 12 mg/kg xylazine. A small incision was made in the dorsal skin and the mice were manipulated to bring the left kidney in close proximity to the body wall. One million 786-O-Fluc or 0.5 × 10^6^ OSRC2-Fluc cells in 20 µL PBS were then injected into the parenchyma of the left kidney using a 27-gauge needle and 0.3-mL syringe. The skin incision was closed with wound clips. Meloxicam was administered by subcutaneous injection before animals regained consciousness. Anesthetized mice were kept on a warm pad to maintain body temperature throughout the procedure. Mice were given water supplemented with Baytril for 10 d following the procedure. Wound clips were removed 7 d after surgery. Mice were monitored daily for signs of distress.

Tumor growth was monitored by weekly BLI beginning 1 to 2 wk after cell injections. Once tumors were established, as determined by an increasing BLI signal for at least 2 consecutive weeks, tumor-bearing mice were randomized to receive 30 mg/kg PT2399 (formulated in 10% ethanol, 30% PEG400, and 60% water containing 0.5% methylcellulose and 0.5% Tween 80) or vehicle alone daily by oral gavage. Two mice per arm were killed by CO_2_ asphyxiation after 5 d of dosing. Tumors were fixed in 10% paraformaldehyde for 24 h and then switched to 70% ethanol before embedding in paraffin blocks. Suppression of tumor growth and HIF2α activity was assessed by immunohistochemical staining for Ki67 and Cyclin D1, respectively, as described in ref. 43. For end-point studies, after 28 d of dosing, animals were killed by CO_2_ asphyxiation and tumors were harvested, weighed, and fixed in 10% paraformaldehyde for histological analysis.

### Statistical Methods.

Statistical tests were applied as indicated in the figure legends and text and were run using GraphPad Prism software. *P* values < 0.05 were considered significant.

## Supplementary Material

Supplementary File

## Data Availability

The plasmids reported in this article have been deposited in Addgene. All study data are included in the article and/or *SI Appendix*.
