# Supplementary material for: Sensitivity of VHL mutant kidney cancers to HIF2 inhibitors does not require an intact p53 pathway
Source: Proc Natl Acad Sci U S A. 2022 Mar 31;119(14):e2120403119. doi: 10.1073/pnas.2120403119 (PMC9168943; doi:10.1073/pnas.2120403119)
Supplement: Supplementary File [file pnas.2120403119.sapp.pdf]

PNAS
www.pnas.org

Supplementary Information for

Sensitivity of *VHL* Mutant Kidney Cancers to HIF2 Inhibitors Does Not Require an Intact p53 Pathway.

Laura A. Stransky, Sean M. Vigeant, Bofu Huang, Destiny West, Thomas Denize, Emily Walton, Sabina Signoretti, William G. Kaelin Jr.

William G. Kaelin Jr.
Email: William_Kaelin@dfci.harvard.edu

This PDF file includes:

Figures S1 to S6
Tables S1 to S3

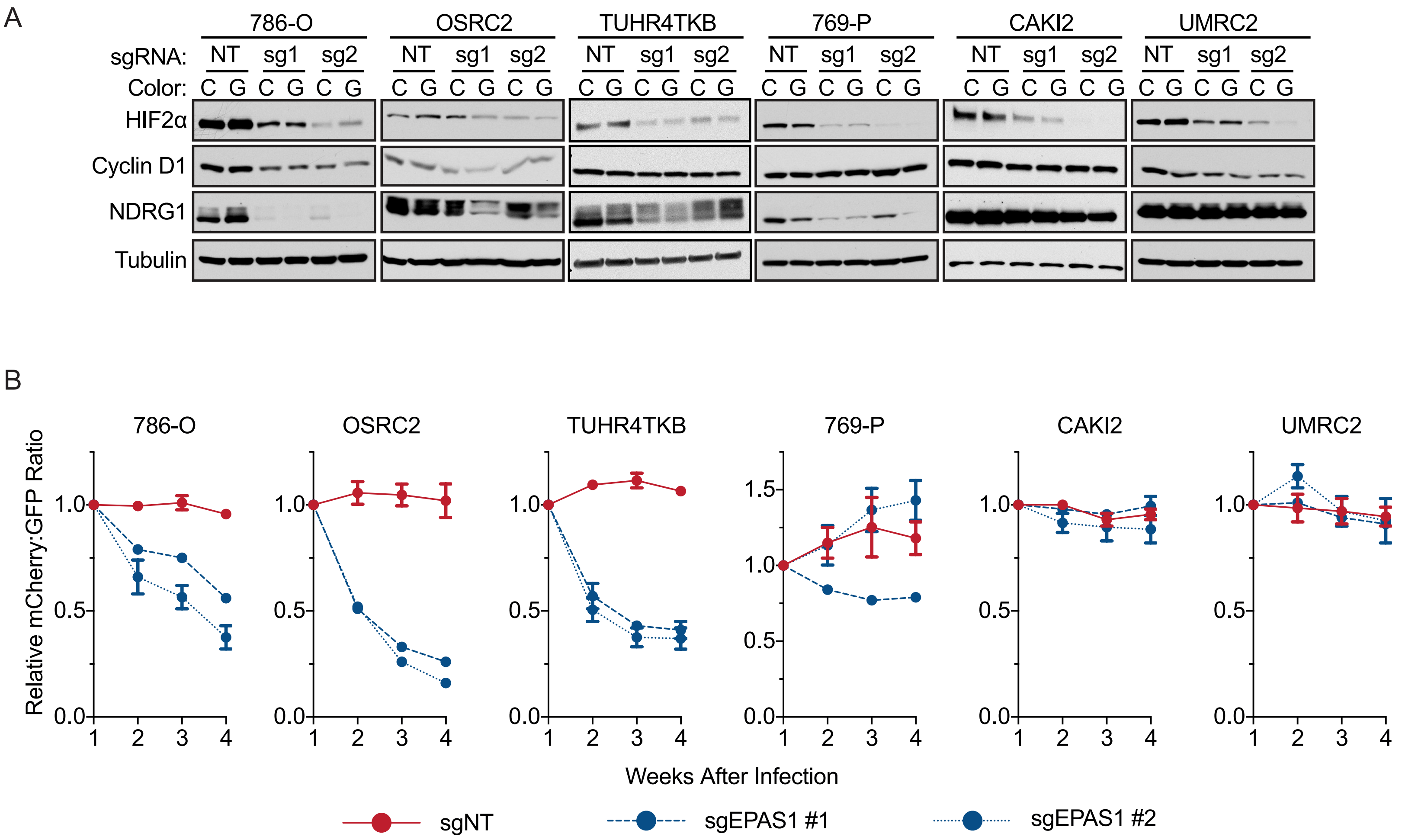

Supplemental Figure 1. HIF2α-Dependence Varies Across VHL Mutant ccRCC Cell Lines.
A. Immunoblots of the indicated VHL mutant ccRCC cell lines engineered to express mCherry (C) or GFP (G) and either a non-targeting control sgRNA (NT) or the indicated sgRNA targeting EPAS1 (sg1 or sg2). B. Relative ratios of mixtures of cell expressing 1) mCherry and either a non-targeting control sgRNA (sgNT) or the indicated sgRNA targeting EPAS1 or 2) GFP and sgNT, over time, as determined by flow cytometry. Ratios were normalized such that the ratio a T = 1 week after lentiviral introduction of the reporters and sgRNAs was 1. Data represents mean +/- SEM of at least 2 independent replicates.

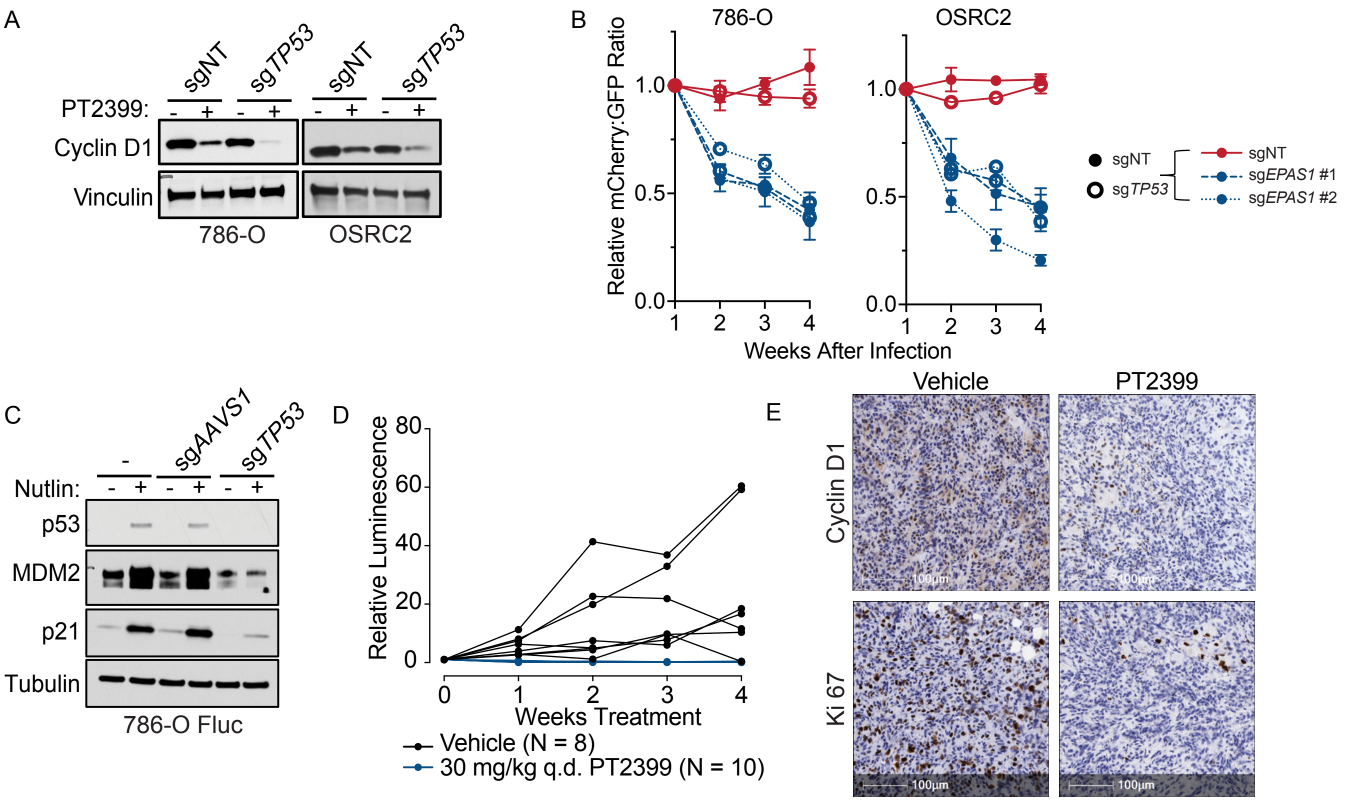

Supplemental Figure 2. Wild-type TP53 is Not Required for HIF2α-Dependence of VHL Mutant ccRCC Lines.

A. Immunoblots of isogenic pairs of 786-O and OSRC2 cells that underwent CRISPR/Cas9-based gene editing with the indicated sgRNAs and then treatment with 2 µM PT2399 or DMSO vehicle control for 24 hours. B. Relative ratio of mixtures of cells from (Fig. 3B) (filled circles = cell expressing non-targeting sgRNA; open circles = cells expressing sgTP53) that were then engineered to express 1) mCherry and either a non-targeting control sgRNA (sgNT) or the indicated sgRNA targeting EPAS1 or 2) GFP and sgNT, over time, as determined by flow cytometry. Ratios were normalized such that the ratio at T = 1 week after lentiviral introduction of the reporters and sgRNAs. Data represents the mean +/- SEM of at least two independent replicates. C. Immunoblot of 786-O cells engineered to express firefly luciferase (786-O Fluc) that underwent CRISPR/Cas9-based gene editing with the indicated sgRNAs (sgAAVS1 = negative control sgRNA) and then treatment, where indicated, with 10 µM Nutlin or DMSO vehicle control for 24 hours. D. Relative BLI intensity over time in individual orthotopic xenografts (left kidney) formed by 786-O that stably expressed firefly luciferase and in which TP53 was inactivated using CRISPR/Cas9 (sgTP53 #1). The tumor-bearing mice were treated daily with 30 mg/kg PT2399 or vehicle control by oral gavage. For each mouse BLI readings were normalized to the pretreatment BLI value for that mouse. E. Immunohistochemical staining for cyclin D1 and Ki67 in orthotopic 786-O Fluc sgTP53 xenografts from mice treated with 30 mg/kg QD PT2399 or vehicle control for 5 days.

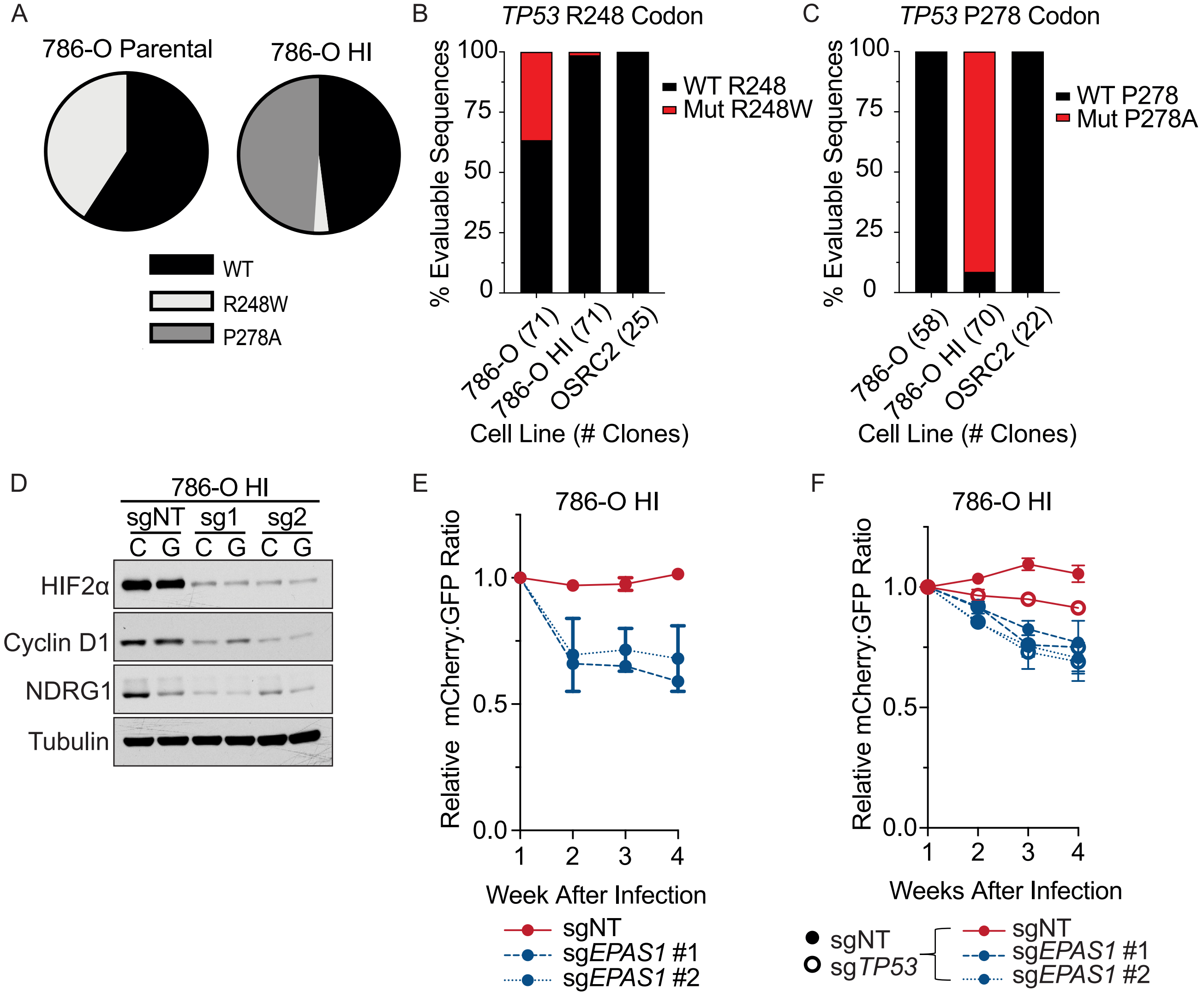

Supplemental Figure 3. TP53 Mutation is not Required for the HIF2α-Independence of the 786-O HI Cells.
A. Proportions of TP53 genomic locus sequencing reads from 786-O parental and 786-O HI cells carrying the indicated TP53 mutations as determined by next generation whole exome sequencing. B. Proportion of individual TP53 cDNAs from 786-O parental, 786-O HI, and OSRC2 carrying the indicated codon 248 sequences as determined by Sanger sequencing. The numbers in parentheses indicate the total number of cDNAs with evaluable sequences for codon 248. C. Proportion of individual TP53 cDNAs from 786-O parental, 786-O HI, and OSRC2 carrying the indicated codon 278 sequences as determined by Sanger sequencing. The numbers in parentheses indicate the total number of cDNAs with evaluable sequences for codon 278. D. Immunoblots of the 786-O HI cells engineered to express Cas9 and then to express mCherry (C) or GFP (G) with either a non-targeting control sgRNA (sgNT) or the indicated sgRNA targeting EPAS1 (sg1 or sg2). E. Relative ratios of mixtures of 786-O HI cells (as in in D) expressing 1) mCherry and either a non-targeting control sgRNA (sgNT) or the indicated sgRNA targeting EPAS1 or 2) GFP and sgNT, over time, as determined by flow cytometry. Ratios were normalized such that the ratio at T = 1 week after lentiviral introduction of the reporters and sgRNAs was 1. Data represents the mean +/- SEM of at least two independent replicates. F. Relative ratio of mixtures of 786-O HI cells (filled circles = cell expressing non-targeting sgRNA; open circles = cells expressing sgTP53) that were then engineered to express 1) mCherry and either a non-targeting control sgRNA (sgNT) or the indicated sgRNA targeting EPAS1 or 2) GFP and sgNT, over time, as determined by flow cytometry. Ratios were normalized such that the ratio at T = 1 week after lentiviral introduction of the reporters and sgRNAs was 1. Data represents the mean +/- SEM of at least two independent replicates.

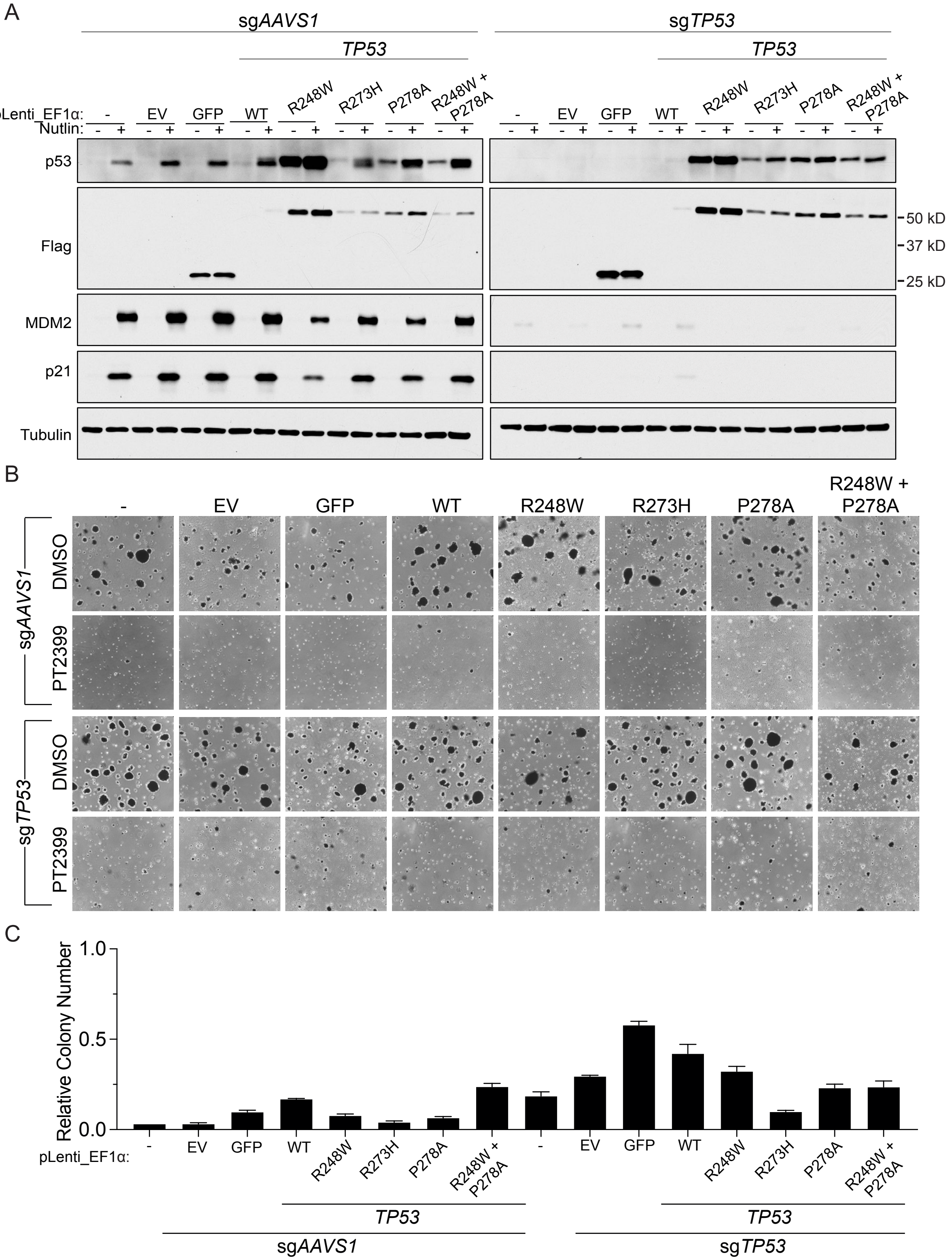

Supplemental Figure 4. Expression of TP53 Mutations Previously Associated with HIF2α-Independence is Not Sufficient to Confer HIF2α-Independence.

A. Immunoblots of OSRC2 cells expressing control sgRNA (sgAAVS1) or sgTP53 that were then engineered to express GFP, the indicated p53 variants, or an empty vector (EV). The cells were (+) or were not (-) treated with 10 μM Nutlin for 24 hours prior to harvest. B. Representative photomicrographs of soft agar colonies formed by OSRC2 cells as in (A) that were grown in the presence of 2 μM PT2399 or DMSO vehicle control. C. Quantification of soft agar colonies formed by OSRC2 cells as in (B). Shown are mean colony numbers in the presence of 2 μM PT2399 relative to DMSO. Data are mean +/- SEM of at least two independent replicates.

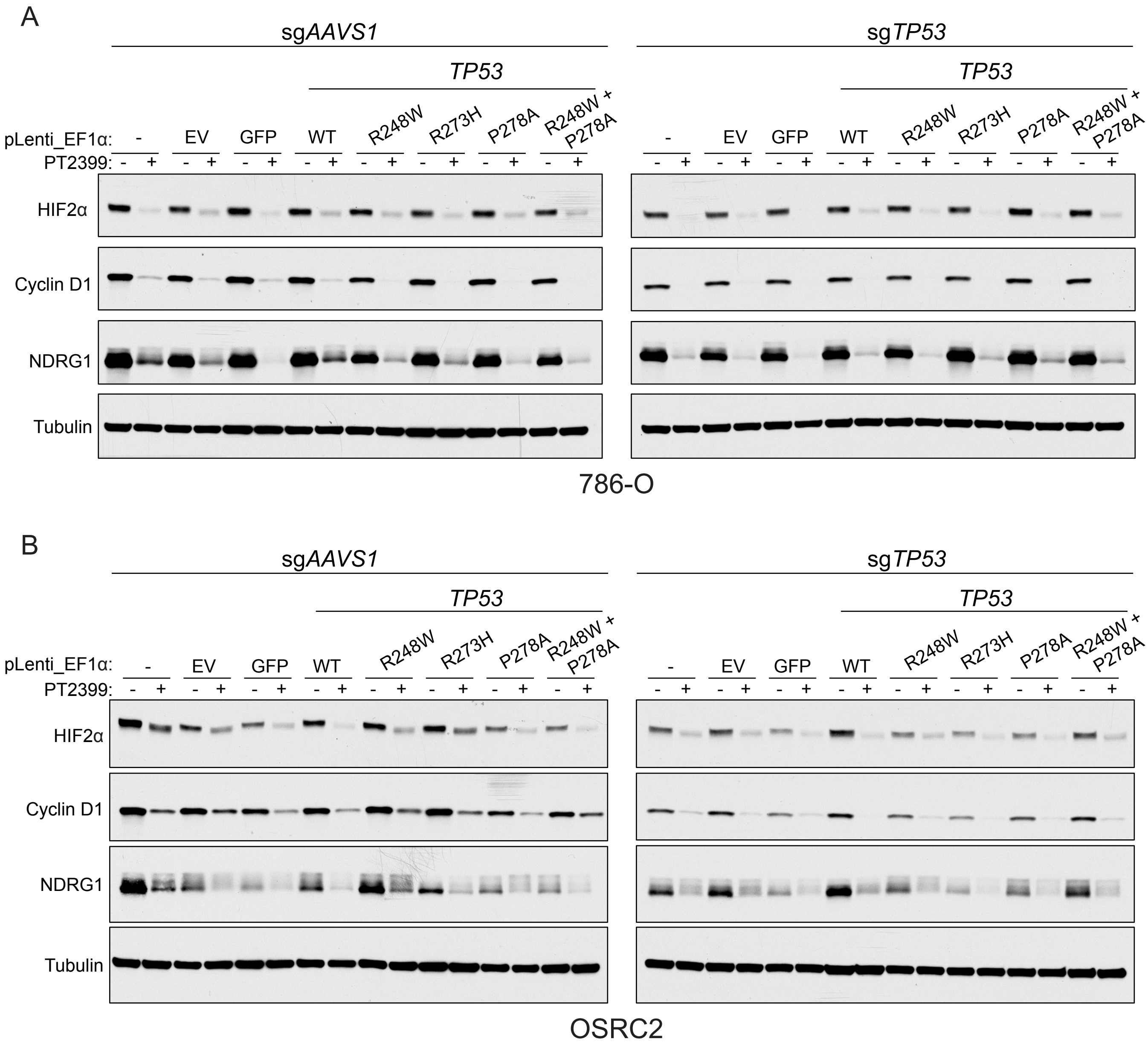

Supplemental Figure 5. Pharmacodynamic Effect of PT2399 is Not Altered by p53 Status. A. Immunoblots of 786-O cells expressing control sgRNA (sgAAVS1) or sgTP53 that were then engineered to express GFP, the indicated p53 variants, or an empty vector (EV). The cells were (+) or were not (-) treated with 2 μM PT2399 for 24 hours prior to harvest. B. Immunoblots of OSRC2 cells expressing control sgRNA (sgAAVS1) or sgTP53 that were then engineered to express GFP, the indicated p53 variants, or an empty vector (EV). The cells were (+) or were not (-) treated with 2 μM PT2399 for 24 hours prior to harvest.

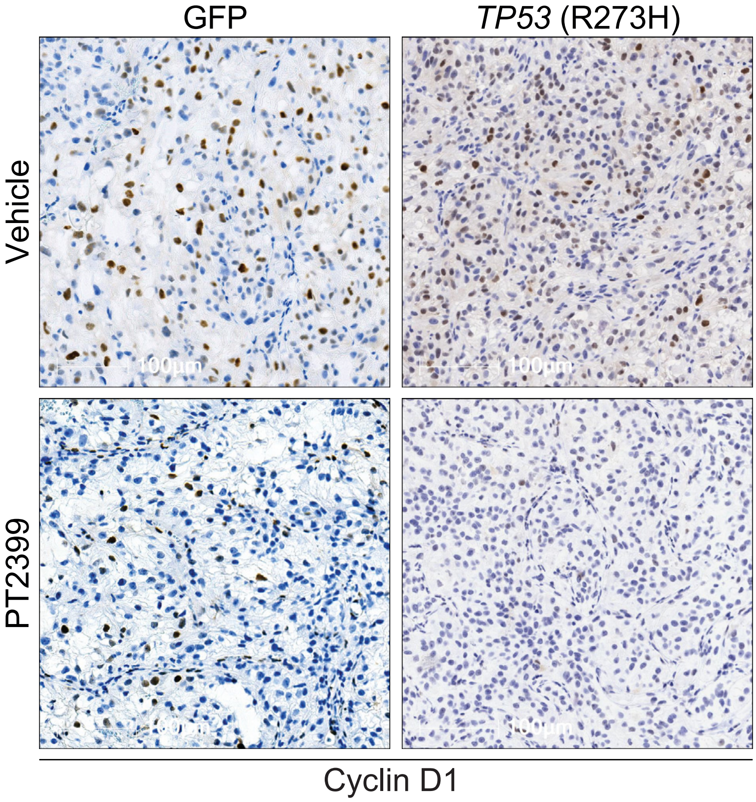

Supplemental Figure 6. Expression of the TP53 R273H Mutation Previously Associated with HIF2α-Independence Does Not Alter PT2399 Pharmacodynamic Effect
Immunohistochemical staining for cyclin D1 in orthotopic OSRC2-Fluc expressing GFP *TP53* R273H (as in Fig. 6a) xenografts from mice treated with 30 mg/kg PT2399 or vehicle control daily for 5 days.

Sample	Designation	D5S818	D13S317	D7S820	D16S539	vWA	TH01	AMEL	TPOX	CSF1PO
786-O (ATCC)	Reference	9	8	11, 12	12	15, 17	6, 9.3	X, Y	8, 11	10
786-O Parental	Test	9	8	11, 12	12	15,17	6, 9.3	X, Y	8, 11	10
786-O HI	Test	9	8	11, 12	12	15, 17	6, 9.3	X, Y	8, 11	10

Supplemental Table 1. STR analysis of 786-O Parental and 786-O HI Cell Lines.

Cell	Chr 1	Chr 2	Chr 3	del(3p)	Chr 4	add(4q)	Chr 5	Chr 6	Chr 7	add(7p)	i(7q)	Chr 8	i(8q)	Chr 9	add(9p)	i(9q)	Chr 10	add(10p)	Chr 11	add(11p)	Chr 12	del(12q)	Chr 13	Chr 14	Chr 15	Chr 16	Chr 17	i(17q)	Chr 18	Chr 19	Chr 20	Chr 21	Chr 22	X Chr	Y Chr	markers	Total
1	3	4	3	1	1	0	2	2	1	1	0	1	0	0	0	1	2	0	2	0	2	0	2	2	3	3	2	0	2	2	3	3	2	1	0	4	55
2	3	2	3	0	2	0	2	2	3	0	1	2	1	0	0	2	2	0	2	1	4	0	2	2	3	0	2	0	1	2	3	3	3	2	1	4	60
3	3	4	0	1	1	1	2	1	4	0	0	2	1	1	0	1	3	1	2	0	3	0	3	1	2	3	1	0	2	2	2	2	3	2	1	2	57
4	4	4	3	1	1	2	2	3	4	0	1	2	2	1	0	2	4	0	2	1	4	0	2	2	3	3	3	0	3	3	3	4	4	2	3	4	82
5	4	3	3	1	2	1	2	3	3	1	0	2	1	1	0	2	4	0	3	0	3	0	3	2	3	3	3	1	3	2	4	5	2	2	2	4	78
6	4	4	3	1	1	2	2	3	4	1	0	2	3	1	0	1	4	0	3	0	4	0	3	2	3	3	3	0	3	3	3	3	4	2	2	8	85
7	4	3	3	1	1	2	2	3	4	0	1	2	2	1	0	2	4	0	3	0	3	0	3	2	3	4	3	0	3	3	4	3	3	1	2	4	79
8	2	4	3	1	2	1	2	3	4	1	0	2	1	1	0	1	4	0	3	0	2	1	3	2	3	3	3	0	3	3	4	3	3	2	3	9	82
9	3	3	3	1	2	1	1	3	3	1	0	1	1	1	0	1	3	0	2	0	2	1	3	1	2	3	3	0	1	2	4	3	3	2	1	10	71
10	4	4	3	1	1	1	2	3	4	1	0	2	1	1	0	2	4	0	3	0	4	0	3	2	3	3	3	0	3	3	3	4	2	2	2	9	83
11	3	4	3	1	1	2	2	3	4	0	1	2	2	1	0	2	3	0	4	0	3	0	3	2	3	2	3	0	2	2	4	2	3	2	1	8	78
12	4	4	3	1	1	2	2	3	3	1	1	2	2	1	0	2	4	0	3	0	4	0	3	3	3	4	3	0	3	3	4	4	3	2	2	3	83
13	2	2	3	0	2	0	2	2	3	1	0	1	1	1	0	2	4	0	0	0	3	0	3	2	1	3	2	0	3	2	4	4	1	2	1	5	62
14	3	4	3	1	2	1	2	3	3	1	0	2	1	1	1	1	4	0	3	0	2	1	3	2	2	3	3	0	3	3	4	3	3	2	2	9	81
15	4	2	0	0	1	1	2	3	2	1	0	2	1	1	0	1	4	0	3	0	3	0	3	1	1	4	2	0	2	2	4	3	3	2	1	4	63
16	3	3	4	1	2	1	2	2	4	1	0	2	1	1	0	1	4	0	3	0	3	0	3	2	3	3	4	0	3	3	4	3	2	2	3	9	82
17	3	4	3	1	1	2	2	3	2	1	1	2	2	2	0	1	4	0	3	0	4	0	2	2	3	4	3	0	3	2	3	5	4	1	1	6	80
18	3	4	3	1	2	1	2	3	4	1	0	2	1	0	1	2	4	0	3	0	1	1	3	3	3	3	3	0	3	3	4	3	3	2	3	8	83
19	4	4	3	1	1	2	2	3	4	0	1	2	2	1	0	1	3	0	3	0	2	0	3	3	3	3	3	0	3	3	3	4	4	2	3	8	84
20	3	4	3	1	2	1	2	3	4	1	0	3	1	1	0	1	4	0	3	0	3	0	3	2	3	3	3	0	3	3	4	3	3	2	3	9	84
21	4	4	3	1	2	0	1	1	3	0	1	1	1	1	0	2	2	0	2	0	4	0	3	1	3	3	2	0	3	3	2	3	4	1	2	5	68
22	4	4	3	1	2	1	2	3	3	1	0	2	1	1	0	2	3	0	3	0	3	0	3	2	3	3	3	1	3	4	3	4	2	2	1	6	79
23	4	3	3	1	1	2	2	4	3	0	1	2	2	1	0	2	4	0	3	0	4	0	3	2	3	3	3	0	3	2	4	4	4	2	2	5	82
24	4	4	3	1	1	2	1	2	3	1	1	1	2	1	0	2	2	1	2	0	3	0	2	2	3	3	2	0	2	3	3	2	3	2	2	4	70
25	2	2	2	1	1	1	1	1	4	1	0	1	0	0	0	2	1	1	2	0	3	0	1	2	1	3	2	0	2	2	1	3	3	1	0	3	50

Key

Chr chromosome
i isochromosome
del partial deletion
add addition of chromatin
p short arm of chromosome
q long arm of chromosome

Supplemental Table 2. Karyotypes of 25 786-O Parental Cells.

Cell	Chr 1	i(1p)	Chr 2	Chr 3	del(3p)	Chr 4	add(4q)	Chr 5	Chr 6	Chr 7	add(7p)	Chr 8	i(8q)	Chr 9	i(9q)add(q)	Chr 10	Chr 11	Chr 12	Chr 13	i(13q)	Chr 14	Chr 15	Chr 16	Chr 17	i(17q)	Chr 18	Chr 19	Chr 20	Chr 21	Chr 22	X Chr	Y Chr	markers	Total	Comments
1	1	0	3	3	1	2	1	2	3	2	0	0	1	0	0	3	3	3	0	1	3	3	1	2	0	3	1	1	3	1	1	1	5	54	
2	4	2	4	4	0	0	3	2	5	5	1	3	3	0	3	2	4	2	0	2	6	2	3	3	0	3	4	1	5	5	1	0	15	97	
3	4	1	2	4	0	2	2	2	4	4	1	2	2	0	2	3	3	3	1	1	4	2	2	4	0	3	3	2	4	4	0	0	13	84	
4	3	0	3	4	0	2	2	2	4	4	0	1	2	0	2	4	3	3	2	0	3	3	3	3	0	3	3	2	3	3	2	1	10	80	
5	3	1	4	4	0	2	2	2	4	5	0	2	2	0	2	4	4	4	2	1	3	3	2	4	0	3	2	2	2	4	1	0	8	82	
6	3	1	1	4	0	2	2	2	4	3	1	2	2	0	2	2	4	2	0	0	3	2	3	4	0	3	4	2	3	4	1	0	7	73	
7	3	1	4	3	0	2	2	2	4	5	0	2	2	0	2	1	4	3	1	1	3	3	3	4	0	3	3	2	4	4	2	0	8	81	
8	3	1	4	3	1	2	2	2	4	4	0	2	2	0	2	3	4	3	1	1	3	3	3	5	0	3	3	2	4	4	1	0	7	82	
9	3	1	4	4	0	2	2	2	3	5	0	2	2	0	1	2	4	3	1	1	3	3	3	5	0	3	3	2	4	4	1	0	9	82	
10	3	0	2	1	0	0	1	2	3	2	0	1	1	0	1	1	4	1	0	1	3	1	3	1	0	2	2	1	2	1	1	0	8	49	
11	3	1	4	4	0	2	2	2	4	5	0	2	2	0	2	3	3	3	1	1	2	3	3	5	0	3	4	2	3	4	1	0	8	82	
12	3	1	4	4	0	2	2	2	4	5	0	2	2	0	2	4	4	3	1	1	4	3	3	4	0	3	3	3	4	4	1	0	6	84	
13	3	1	3	4	0	2	2	2	4	5	0	2	2	0	1	4	4	3	1	1	4	3	3	4	0	2	3	2	4	4	1	0	10	84	
14	3	1	3	4	0	2	2	2	4	5	0	2	2	0	1	3	4	3	1	1	3	3	2	4	0	3	4	3	4	4	1	0	8	82	
15	3	1	3	4	0	2	2	2	4	6	0	2	2	0	2	3	4	3	1	1	3	3	3	5	0	3	3	2	4	4	1	1	8	85	
16	3	1	4	4	0	2	2	2	4	5	0	2	2	0	2	4	4	3	1	1	3	3	3	4	0	4	3	2	4	3	1	0	6	82	
17	3	1	4	4	0	2	2	2	4	5	0	1	2	0	1	4	4	3	1	1	3	3	3	4	0	3	3	3	4	4	1	0	8	83	
18	1	0	3	1	0	1	2	0	1	3	0	1	2	0	0	4	3	2	1	1	2	3	3	3	0	3	3	2	3	2	1	0	7	58	
19	3	0	3	4	0	1	0	2	4	3	0	2	2	0	1	2	4	2	1	1	3	2	3	4	0	3	4	2	4	4	2	0	8	74	One Chr3 has add(p), two markers are dicentrics
20	2	1	4	4	0	2	2	2	4	5	0	1	2	0	2	1	4	3	1	1	3	3	2	3	0	3	3	2	4	4	1	0	11	80	
21	3	1	4	4	0	2	2	2	4	5	0	2	2	0	2	3	4	3	1	1	3	3	3	4	0	3	4	2	4	4	1	0	9	85	
22	4	0	3	4	0	2	2	2	3	4	0	2	2	1	1	3	4	3	2	0	4	3	3	4	0	2	4	2	4	4	1	0	9	82	"normal" i(9q)
23	3	1	4	4	0	2	2	2	3	4	0	2	1	0	2	3	4	3	1	1	2	3	3	4	0	3	4	2	4	4	1	0	9	81	
24	2	1	4	3	0	2	2	2	4	5	0	2	2	0	2	3	4	2	1	1	3	3	3	4	0	3	4	2	4	4	1	0	9	82	
25	3	1	4	4	0	2	2	2	4	4	0	2	2	0	2	3	4	2	1	1	3	3	3	4	0	3	4	2	4	4	1	0	10	84	One Chr 10 has add(10q), dicentric 10;12

Key

Chr chromosome
i isochromosome
del partial deletion
add addition of chromatin
p short arm of chromosome
q long arm of chromosome

Supplemental Table 3. Karyotypes of 25 786-O HI Cells.
